# Association between anemia and serum Klotho in middle-aged and older adults

**DOI:** 10.1186/s12882-023-03081-w

**Published:** 2023-02-16

**Authors:** Chencheng An, Xiaoling Chen, Donghui Zheng

**Affiliations:** Department of Nephrology, Huai’an Hospital Affiliated to Xuzhou Medical University and Huai’an Second People’s Hospital, Huaian, 223002 Jiangsu China

**Keywords:** Anemia, NHANES, Klotho

## Abstract

**Background:**

The role of Klotho as a multifunctional protein in anemia is unclear. This study aimed to determine the association between anemia and serum Klotho concentrations in middle-aged and elderly populations.

**Methods:**

In this cross-sectional study, we used data collected from the National Health and Nutrition Examination Survey (NHANES) 2007-2016. A total of 13,357 individuals who received serum Klotho measurements, biochemical tests, and demographic surveys were analyzed. Multivariate linear regression models adjusting for covariates were used to investigate the associations between anemia and serum Klotho.

**Results:**

Multivariable regression showed that serum Klotho correlates positively with hemoglobin and red blood cells and inversely with red cell distribution width. After adjusting for all covariates, compared with Q4, there was a significantly increased risk of anemia in serum Klotho quartiles 1 to 2 (OR=1.54, 95% CI:1.21-1.95, P=0.002; OR=1.30, 95% CI:1.02-1.64, P=0.042,respectively). Segmented regression showed that for every 100 pg/mL increase in serum Klotho <9.746 pg/mL, the risk of anemia was reduced by 10.9%, and this reduction was significant (P<0.001). Furthermore, stratified analyses yielded a stronger association between reduced anemia and high levels of Klotho in men and those with diabetes (P< 0.05 for interaction). However, this association was not found to be significantly altered by chronic kidney disease.

**Conclusions:**

In summary, we indicated that low serum Klotho is associated with an increased likelihood of anemia using a nationally representative sample of middle-aged and older adults.

**Supplementary Information:**

The online version contains supplementary material available at 10.1186/s12882-023-03081-w.

## Introduction

According to data reported in the Global Burden of Disease Study 2019, approximately 1.8 billion people worldwide suffer from anemia [1]. Anemia is a global public health problem and a heavy health burden worldwide, caused by nutritional (iron, vitamin B12, folic acid deficiency) or non-nutritional (reduced hematopoietic tissue, inflammation, infection, chronic disease, or immune decompensation) factors. Anemia in the elderly is usually slow and insidious, with less than 1% of the elderly population being moderately severely anemic, and men $>=$75 years are more likely to be anemic than women [2]. Aging contributes to defects in the hematopoietic reconstitution function of hematopoietic stem cells (HSC), including reduced self-renewal viability, altered differentiation potential (decreased lymphoid progenitor cells), DNA damage, and changes in signaling pathways. Anemia in middle-aged and older people is often associated with negative outcomes, including reduced physical function and significantly worse survival rate [3, 4].

KLOTHO has been identified as a senescence suppressor gene encoding the $\alpha$-Klotho, $\beta$-Klotho, and $\gamma$-Klotho proteins. High levels of $\alpha$-Klotho are measured in the kidney and brain and the transmembrane and soluble secreted form of $\alpha$-Klotho (S-Klotho) is detectable in humans and mice [5]. Since the identification of this gene in 1997, it has been recognized that the Klotho protein acts as a membrane receptor binding to the fibroblast growth factor receptor (FGFR) to regulate the mineral metabolism. Klotho can also act as a circulating hormone or paracrine factor affecting other organs such as the brain, adipose tissue, and skeletal muscle, performing many housekeeping functions in almost every organ [6]. However, the role of $\alpha$-Klotho in hematopoietic tissues is unclear. Studies in mice have shown that klotho is involved in the regulation of iron metabolism [7] and hematopoiesis [8]. It has been shown that in patients with chronic kidney disease, low levels of S-Klotho are associated with an increased risk of developing anemia in patients [8]. Conversely, it has also been reported that soluble Klotho has not been found to be associated with hemoglobin [9]. A limitation of these surveys is the small sample of people enrolled.

The relationship between anemia and serum klotho is complex and we are the first research to use large data on the relationship in the general middle-aged and elderly population.

## Material and method

### Study population

The National Health and Nutrition Examination Survey (NHANES) is a continuous survey (1999-present) designed to measure noninstitutionalized civilian US residents’ health and nutritional status. In NHANES, a standardized questionnaire was administered in the home by a trained interviewer and a physical examination was conducted at a mobile examination center. It was approved by the NCHS Research Ethics Review Board [10]. Data user agreements were available online for all cycles of the NHANES Informed consent or Institutional Review Board approval is not required for analysis of NHANES data, which are all de-identified. Available at: https://www.cdc.gov/nchs/nhanes/hlthprofess.htm.

**Fig. 1:**
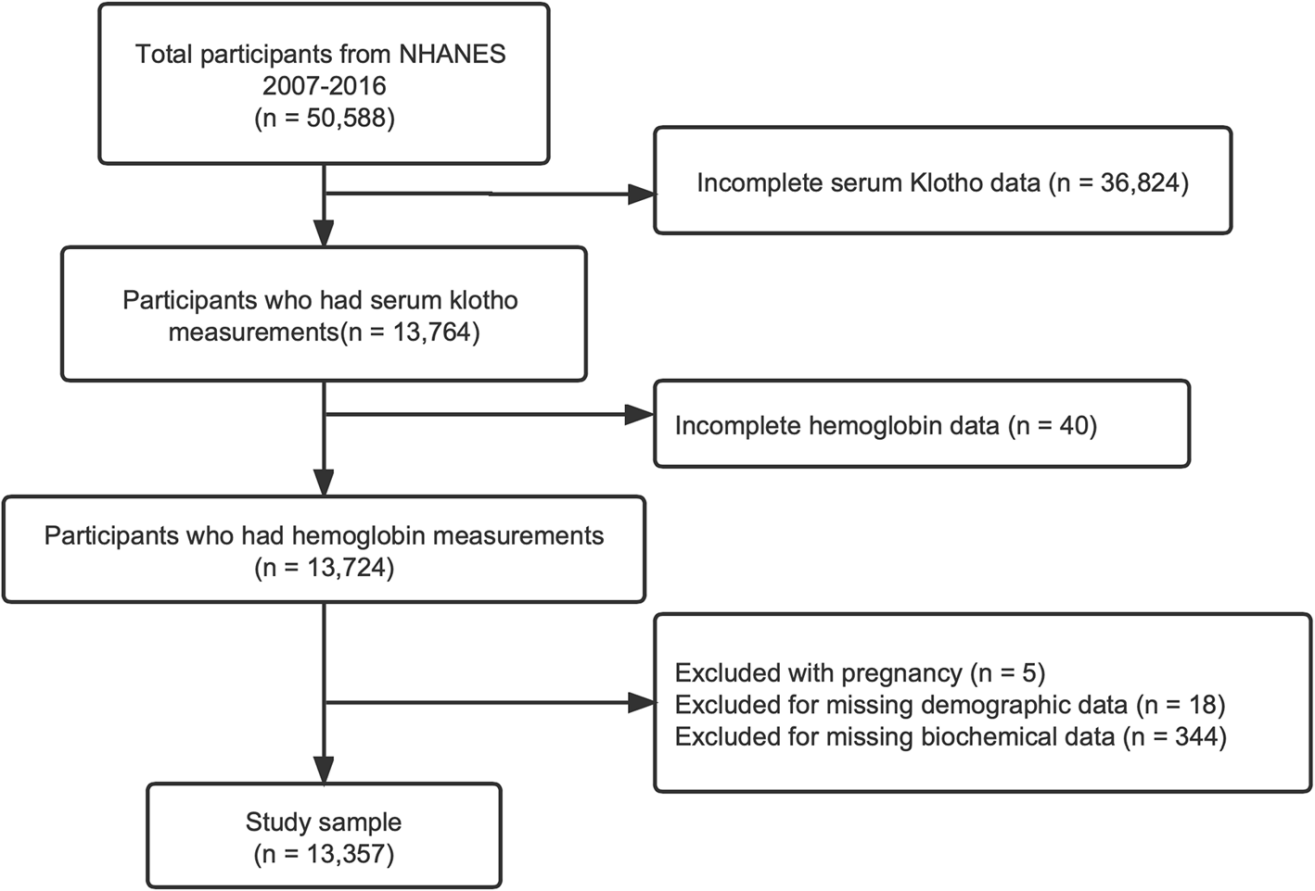
Eligible participants in the evaluation of the association between anemia and S-Klotho in middle-aged and elderly individuals

We assessed subpopulations of middle-aged and elderly from five cycles of the NHANES 2007-2008, 2009-2010, 2011-2012, 2013-2014, and 2015-2016 ($n = 50,588$). This study included volunteers who had serum Klotho measurements, and who had hemoglobin data ($n = 13,724$). Pregnant women were excluded ($n = 5$). In addition, we excluded samples with missing demographic ($n =18$) and biochemical data ($n = 344$). A total of 13,357 respondents were eventually considered (Fig. 1).

### Study variables

Serum Klotho

Original blood serum samples from volunteers aged from 40 to 79 years in NHANES 2007-2016. Study participants gave consent to the storage of blood samples and the use of blood for future research. All samples were stored at -80$^{\circ }$C before experiments. Serum Klotho was measured using a commercially available ELISA kit $\left( {IBL \; International,Japan} \right)$ following the manufacturer’s instructions and was run in duplicate.

Socio-demographic characteristics

Age, sex, race, and educational attainment. Marital status was classified into 2 groups: having a partner (married, having a partner) and others (never married, separated, divorced, and widowed). We assessed self-reported income by comparing it with the US poverty level (poverty income ratio) and had three levels: PIR < 1.3, near poor 1.3 $<=$ PIR< 3.0, and non-poor PIR $>=$ 3.0. Alcohol use was assessed based on the responses to the following questions: “Had at least 12 alcohol drinks/1 yr?” Smoking status was classified into never, former, and now. Metabolic Equivalents (METs) are used to calculate the total physical activity MET-min/week and are divided into three categories: inactive (MET < 600), moderate-to-vigorous physical activity (MET $>=$ 600), or missing physical activity data.

Anthropometric Measures

Body mass index (BMI) was calculated as weight in kilograms divided by the measured height in meters squared and was categorized into <20.0,20.0-24.9, 25.0-29.9, or$>=$ 30.0 kg/m2. Waist circumference $>=$ 102 cm in men or 88 cm in women was high.

Laboratory Measurements

Participants were examined for the analysis of phosphorus, calcium, serum iron, albumin, uric acid, urinary albumin/creatinine ratio, serum 25-hydroxyvitamin D, and creatinine. Serum 25-hydroxyvitamin D = 25-hydroxyvitamin D2 + 25-hydroxyvitamin D3. The estimated glomerular filtration rate (eGFR) was calculated according to Chronic Kidney Disease Epidemiology Collaboration (CKD-EPI Cr) equation.

Dietary intake

Detailed information on dietary intake was obtained personally by participants during the first dietary recall interview (MEC), estimating total energy, protein, DFE (folic acid as dietary folate equivalent, $\upmu$g), vitamin B12 ($\upmu$g), and iron intake (mg) from foods and beverages consumed in the 24 hours before the interview. We consider DFE deficiency as less than 400 mcg, vitamin B12 deficiency as less than 2.4 mcg, and iron intake deficiency as less than 12 mg [11].

Anemia Diagnosis

According to WHO criteria, the hemoglobin level (g/dl) for diagnosing anemia at sea level is hemoglobin<12 for women; hemoglobin<13 for men. Beckman Coulter HMX instrument in the Mobile Examination Centre (MEC) produces a full blood count on a blood sample and provides blood cell distribution for all attendees.

Comorbidities

Antihypertensive medications, self-reported hypertension, or$>=$140 (systolic blood pressure)/90 (diastolic blood pressure) mmHg were used to diagnose hypertension. Hyperlipidemia was defined as patients on lipid-lowering medication or with total cholesterol $>=$ 220 mg/dl, HDL < 40 mg/dl (men), or HDL < 50 mg/dl (women), TG $>=$150 mg/dl. The verification of angina, congestive heart failure, coronary artery disease, cancer, and rheumatoid arthritis was based on the questionnaire from MCQ. The verification of heart attack and stroke was based on the questionnaire from MCQ or SPQ. Diabetes mellitus(Diabetes) was diagnosed based on (1) self-reported diabetes (2) fasting plasma glucose $>=$ 126 mg/dL (3) random blood glucose $>=$ 200 mg/dL (4) two-hour postprandial blood glucose (2hPBG) $>=$ 200 mg/dL based on oral glucose tolerance test (OGTT 75 grams) (5) glycated hemoglobin A1c $>=$ 6.5% or (6) current use of anti-diabetic medications (oral hypoglycemic medications or insulin). Chronic kidney disease (CKD) was defined as eGFR <60 ml/min per 1.73 m2 or UACR $>=$30 mg/g.

### Statistical Analyses

Based on guidelines from the National Center for Health Statistics (NCHS), we used sample weights, primary sampling units, and strata in our analyses to account for the complex sampling design.

The population is characterized according to the presence or absence of anemia. The descriptive statistics presented here summarize categorical variables with frequencies and percentages, while continuous variables are summarized with medians [interquartile range (IQR)]. Weighted logistic regression models were used for multivariate analyses. Owing to numerous potential confounders, we created 3 adjustment models. Model 1: modified for age, gender, and race. Model 2: further adjusted for marital status(having a partner, others), poverty-to-income ratio(<1.3,1.3-2.9, $>=$3.0), an education level (< high school, high school, > high school), alcohol intake (no, yes), smoke (never, former, now), moderate-vigorous physical activity, body mass index (<25.0,25.0-29.9, $>=$30.0), and high waist circumference. Model 3: further adjusted for phosphorus, calcium, serum iron, albumin, uric acid, urinary albumin/creatinine ratio, estimated glomerular filtration rate, serum 25-hydroxyvitamin D, DFE deficiency, Vitamin B12 deficiency, Iron intake deficiency, angina, heart attack, coronary heart disease, congestive heart failure, stroke, hypertension, hyperlipidemia, diabetes, chronic kidney diseases, cancer, and rheumatoid arthritis. Serum Klotho was treated as a categorical variable (quartiles) for regression analysis, and the highest quartile of the categorical variable was used as a reference. A generalized additive model was also used to visually assess the relationship between the two. The results are presented using a smoothed curve fit graph. Segmented regression analysis was applied to assess the effects of anemia on serum Klotho. We have performed interaction analyses and stratified analyses to investigate whether this correlation differs by age (40-59,60-79), gender, body mass index (<25.0,25.0-29.9, $>=$30.0), diabetes, and CKD.

Statistical analyses were performed using IBM SPSS 26.0 (New York, USA) and EmpowerStats (X &Y Solutions Inc). P $\le$0.05 was considered statistically significant.

## Results

### Characteristics of the research sample

The baseline characteristics of the study population ($n = 13,357$) are shown by the presence or absence of anemia in Table 1. The average age of all respondents was 55 years respectively and 52.27% were female. Subjects with anemia ($n = 1,392$) were more likely to be elderly, with higher levels of education, alcohol consumption, high waist circumference, and obesity. Patients with anemia have low blood albumin, eGFR, serum 25-hydroxyvitamin D, and low energy and protein intake.

**Table 1:** Baseline characteristics of the study participants from NHANES 2007-2016, weighted

		Total ($n = 13, 357$)	Non-Anemia ($n = 11, 965$)	Anemia ($n = 1, 392$)	*P*-value
Age, years, (median, IQR)		55.00 (47.00,64.00)	55.00(48.00, 64.00)	56.00(46.00, 68.00)	0.009
Female, n (%)		6898 (52.27)	6043 (51.16)	855 (66.68)	<0.001
Race, n (%)					<0.001
	Mexican American	2122 (6.68)	1920 (6.49)	202 (9.14)	
	Other Hispanic	1535 (4.70)	1400 (4.60)	135 (5.93)	
	Non-Hispanic White	5761 (73.08)	5417 (74.92)	344 (49.23)	
	Non-Hispanic Black	2617 (9.03)	2040 (7.58)	577 (27.91)	
	Other race	1322 (6.51)	1188 (6.41)	134 (7.79)	
Marital status, n (%)					<0.001
	Having a partner	8660 (70.39)	7872 (71.03)	788 (62.07)	
	Others	4697 (29.61)	4093 (28.97)	604 (37.93)	
PIR, n (%)					<0.001
	<1.3	3735 (16.50)	3254 (15.80)	481 (25.63)	
	$>=$1.3, <3.0	3695 (24.33)	3296 (24.01)	399 (28.55)	
	$>=$3.0	4831 (53.05)	4455 (54.22)	376 (37.83)	
Education level, n (%)					<0.001
	< high school	3738 (16.47)	3284 (15.90)	454 (23.87)	
	high school	2955 (22.09)	2654 (22.19)	301 (20.75)	
	> high school	6664 (61.44)	6027 (61.91)	637 (55.39)	
Alcohol intake, n (%)					<0.001
	no	3637 (21.03)	3132 (20.26)	505 (31.08)	
	yes	8772 (72.80)	8003 (73.71)	769 (60.88)	
Smoking status,n (%)					<0.001
	never	6904 (51.86)	6101 (51.26)	803 (59.72)	
	now	2589 (18.32)	2391 (18.77)	198 (12.52)	
	former	3864 (29.82)	3473 (29.97)	391 (27.76)	
Moderate-vigorous physical activity,n (%)		5003 (41.58)	4569 (41.94)	434 (36.90)	<0.001
Body mass index,kg/m$^{2}$, n (%)					<0.001
	<25.0 kg/m$^{2}$	3154 (24.47)	2802 (24.33)	352 (26.37)	
	25.0-29.9 kg/m$^{2}$	4578 (35.03)	4172 (35.53)	406 (28.50)	
	$\ge$30.0 kg/m$^{2}$	5495 (39.71)	4884 (39.41)	611 (43.60)	
High waist circumference, n (%)		8264 (62.51)	7393 (62.56)	871 (61.85)	<0.001
Phosphorus, mmol/L, (median, IQR)		1.20 (1.10, 1.32)	1.20 (1.10, 1.32)	1.23 (1.10, 1.36)	0.008
Calcium, mmol/L, (median, IQR)		2.35 (2.30, 2.40)	2.35 (2.30, 2.40)	2.33 (2.25, 2.38)	<0.001
Serum iron, umol/L, (median, IQR)		14.70 (11.3, 18.80)	15.00 (11.60, 19.20)	9.00 (5.40, 13.10)	<0.001
Albumin, g/dL, (median, IQR)		4.30 (4.10, 4.50)	4.30 (4.10, 4.50)	4.10 (3.90, 4.30)	<0.001
Uric acid, umol/L, (median, IQR)		321.20 (267.70, 380.70)	321.20 (267.70, 380.70)	309.30 (243.90, 386.60)	0.295
UACR, mg/g, (median, IQR)		6.94 (4.64, 12.54)	6.82 (4.62, 12.19)	8.92 (5.21, 23.28)	<0.001
eGFR, ml/min/1.73m2, (median, IQR)		88.78 (75.24, 100.19)	88.89 (75.79, 99.97)	86.90 (64.86, 104.75)	<0.001
serum 25-hydroxyvitamin D,nmol/L, (median, IQR)		71.00 (53.70,88.40)	71.50 (54.50, 88.70)	61.80 (42.60, 84.40)	<0.001
Serum Klotho, pg/mL, (median, IQR)		798.00 (657.20, 980.20)	800.20 (659.70, 980.70)	761.50 (619.70, 967.20)	0.127
Red blood cell, MillionCells/uL, (median, IQR)		4.63 (4.33, 4.94)	4.66 (4.38, 4.96)	4.08 (3.78, 4.41)	<0.001
Hemoglobin, g/dl, (median, IQR)		14.20 (13.30, 15.10)	14.30 (13.60, 15.20)	11.60 (10.90, 11.90)	<0.001
Mean cell volume, fL, (median, IQR)		90.30 (87.30, 93.30)	90.50 (87.60, 93.40)	85.30 (78.10, 90.60)	<0.001
Mean cell hemoglobin, pg, (median, IQR)		30.80 (29.60, 32.00)	30.90 (29.80, 32.00)	28.40 (25.40, 30.70)	<0.001
Red cell distribution width, (median, IQR)		13.00 (12.50, 13.70)	13.00 (12.50, 13.60)	14.60 (13.40, 16.10)	<0.001
Energy intake, kcal, (median, IQR)		875.00 (3026.00, 4892.00)	3904.00 (3044.00, 4923.00)	3495.00 (2711.00, 4499.00)	<0.001
Protein intake, g, (median, IQR)		152.22(117.29, 195.76)	153.47 (118.11, 197.16)	137.08 (103.14, 176.54)	<0.001
DFE deficiency, (median, IQR)		774 (4.91)	654 (4.67)	120 (8.00)	<0.001
Vitamin B12 deficiency, n (%)		638 (3.85)	549 (3.69)	89 (5.94)	<0.001
Iron intake deficiency, n (%)		639 (4.16)	549 (4.03)	90 (5.80)	0.002
Angina, n (%)		418 (2.69)	352 (2.51)	66 (5.02)	<0.001
Heart attack, %		756 (4.57)	639 (4.40)	117 (6.89)	<0.001
Congestive heart failure, n (%)		513 (2.88)	390 (2.49)	123 (8.05)	<0.001
Coronary heart disease, n (%)		673(4.31)	568 (4.05)	105 (7.74)	<0.001
Stroke, n%		627( 3.53)	505 (3.22)	122 (7.59)	<0.001
Hypertension, n (%)		7185 (48.43 )	6274 (47.58)	911 (59.38)	<0.001
Hyperlipidemia, n (%)		10687(79.94)	9637 (80.27)	1050 (75.64)	0.001
Diabetes, n (%)		3421(19.34)	2898 (18.40)	523 (31.45)	<0.001
Chronic kidney disease, n (%)		2688 (16.10)	2176 (14.91)	512 (31.53)	<0.001
Cancer, n (%)		1559(13.48)	1380 (13.39)	179 (14.56)	0.580
Rheumatoid arthritis, n (%)		943(5.46)	801 (5.18)	142 (9.06)	<0.001

Data in the table: For continuous variables: survey-weighted Median (IQR), *P*-value was by survey-weighted linear regression (svyglm).For categorical variables: survey-weighted percentage, *P*-value was by survey-weighted Chi-square test (svytable).

Abbreviations: *PIR* poverty income ratio, *DFE* Folate as dietary folate equivalents, *eGFR* estimated glomerular filtration rate by Chronic Kidney Disease Epidemiology Collaboration (CKD-EPI), *UACR* urinary albumin/creatinine ratio, *IQR* interquartile range

### Association Between anemia and serum Klotho

The multiple regression analysis shows the OR (95% CIs) for the associations between anemia and serum Klotho in Table 2. When classifying serum Klotho into quartiles, we found that Klotho was not significantly associated with anemia at Q3 (840.8- 935.5 pg/mL) (p > 0.05), while at Q1 (496.7 - 613.9 pg/mL) and Q2 (690.9-763.4 pg/mL) (p < 0.05), the association was significant. Serum Klotho Quartile 1 (OR = 1.54, 95% CI = 1.21, 1.95, $P = 0.002$), Quartile 2 (OR = 1.30, 95% CI = 1.02, 1.64, $P = 0.042$) was significantly associated with anemia in the fully adjusted model 3, compared with Q4. Moreover, in Supplementary Table 1 and 2, we found a positive and significant relationship between hemoglobin(g/dL), red blood cells (MillionCells/uL), and serum Klotho respectively. In addition to this, a negative correlation with red cell distribution width was found at Klotho Q1 and Q2 (Supplementary Table 3). Non-linear relationships between anemia-related indicators (probability of anemia, hemoglobin(g/dL), red blood cells (MillionCells/uL), and red cell distribution width) and S-Klotho were further observed by fully adjusted smoothed curve fitting (Supplementary Fig. 1). Segmented regression showed a turning point value of 9.746 (100pg/mL) for serum Klotho for anemia (Table 3). When serum Klotho < 9.746 per 100 pg/mL, the risk of anemia was reduced by 10.9%, and this reduction was significant (P<0.001); however, when Klotho > 9.746 per 100 pg/mL, this relationship was not significant ($P = 0.298$). This result is the same as the smoothed curve fit.

**Table 2:** Relationship between anemia and S-Klotho (pg/mL) in 3 models, weighted

Exposure	Model 1		Model 2		Model 3	
	OR (95% CI)	*P*-value	OR (95% CI)	*P*-value	OR (95% CI)	*P*-value
S-Klotho categories quartiles (IQR)						
Quartile 1 (496.7, 613.9)	1.47 (1.18, 1.83)	0.001	1.57 (1.26, 1.96)	<0.001	1.54 (1.21, 1.95)	0.002
Quartile 2 (690.9, 763.4)	1.22 (1.00, 1.49)	0.060	1.27 (1.03, 1.56)	0.032	1.30 (1.02, 1.64)	0.042
Quartile 3 (840.8, 935.5)	0.98 (0.79, 1.22)	0.887	1.02 (0.82, 1.27)	0.881	1.03 (0.81, 1.31)	0.834
Quartile 4 (1060.8, 1330.9)	Reference		Reference		Reference	
S-Klotho is divided into quartiles (IQR):						
Quartile 1(496.7, 613.9);Quartile 2(690.9, 763.4);Quartile 3(840.8, 935.5);Quartile 4(1060.8, 1330.9).						

Model 1: Modified for age, gender, and race.

Model 2: Modified for age, gender, race, marital status(having a partner, others), poverty-to-income ratio (<1.3, 1.3-2.9, $>=$3.0), education level (< high school, high school, > high school), alcohol intake (no, yes), smoke (never, former, now), moderate-vigorous physical activity, body mass index (<25.0, 25.0-29.9, $\ge$30.0), and high waist circumference.

Model 3: Modified for age, gender, race, marital status (having a partner, others), poverty-to-income ratio (<1.3, 1.3-2.9, $>=$3.0), education level (< high school, high school, > high school), alcohol intake (no, yes), smoke (never, former, now), moderate-vigorous physical activity, body mass index (<25.0, 25.0-29.9,$\ge$30.0), high waist circumference, phosphorus, calcium, serum iron, albumin, uric acid, urinary albumin/creatinine ratio, estimated glomerular filtration rate, serum 25-hydroxyvitamin D, DFE deficiency, Vitamin B12 deficiency, Iron intake deficiency, angina, heart attack, coronary heart disease, congestive heart failure, stroke, hypertension, hyperlipidemia, diabetes, chronic kidney diseases, cancer, and rheumatoid arthritis.

Abbreviations: *OR* odd ratio, *CI* confidence interval

**Table 3:** Threshold effects of S-Klotho (per 100 pg/mL) for anemia were analyzed in NHANES 2007-2016 using a two-piece regression model

Exposure	anemia, Adjusted OR (95% CI), *P*-value
Fitting by the standard linear model	0.956 (0.936, 0.976) <0.001
Fitting by the two-piecewise linear model	
Turning point (K)	9.746
< Turning point (K)	0.891 (0.857, 0.925) <0.001
>Turning point (K)	1.018 (0.985, 1.052) 0.298
Predicted at Turning point (K)	-2.471 (-2.570, -2.372)
Log likelihood ratio test	<0.001

Adjusted the covariates: age, gender, race, marital status (having a partner, others), poverty-to-income ratio (<1.3,1.3-2.9, $>=$3.0), education level (< high school, high school, > high school), alcohol intake (no, yes), smoke (never, former, now), moderate-vigorous physical activity, body mass index (<25.0, 25.0-29.9,$\ge$30.0), high waist circumference, phosphorus, calcium, serum iron, albumin, uric acid, urinary albumin/creatinine ratio, estimated glomerular filtration rate, serum 25-hydroxyvitamin D, DFE deficiency, Vitamin B12 deficiency, Iron intake deficiency, angina, heart attack, coronary heart disease, congestive heart failure, stroke, hypertension, hyperlipidemia, diabetes, chronic kidney diseases, cancer, and rheumatoid arthritis.

Abbreviations: *OR* odd ratio, *CI* confidence interval

### Stratified analyses by age, gender, BMI, diabetes, and chronic kidney disease

To assess the robustness of the correlation between anemia and serum Klotho, a subgroup analysis was performed. In Table 4, the observed correlation between the two was stronger in men (P for interaction <0.05). When we restricted the analysis to males, the odds of anemia were 2.81, 1.79, and 1.39 for participants within quartiles 1, 2, and 3, respectively, when compared to Klotho quartile 4. We derived that the association between the odds of developing anemia with diabetes and Klotho was stronger (P <0.05 for the interaction). In contrast, the lower the Klotho concentration, the higher the chance of developing anemia. Nevertheless, the association was not distinctly altered by chronic kidney disease.

**Table 4:** Subgroup analyses for the association between anemia and quartiles of S-Klotho (pg/mL) among the study participants, weighted

anemia	S-Klotho Quartile, OR (95% CI)				P-interaction
	Quartile 1 (496.7, 613.9)	Quartile 2 (690.9, 763.4)	Quartile 3 (840.8, 935.5)	Quartile 4 (1060.8, 1330.9)	
Age$^{a}$					0.561
40-59	1.38(1.00, 1.91)	1.17 (0.86, 1.60)	1.00 (0.73, 1.35)	Reference	
60-79	1.85 (1.31,2.62)	1.57 (1.11, 2.21)	1.12 (0.78, 1.62)	Reference	
Gender$^{b}$					0.005
Male	2.81 (1.89,4.19)	1.79 (1.25, 2.58)	1.39 (0.96, 2.03)	Reference	
Female	1.15 (0.85,1.54)	1.17 (0.85, 1.59)	0.93 (0.69, 1.26)	Reference	
Body mass index$^{c}$					0.418
< 25.0 kg/m$^{2}$	1.53 (0.99,2.34)	1.53 (0.94, 2.49)	0.87 (0.56, 1.36)	Reference	
25.0-29.9 kg/m$^{2}$	1.43 (0.88,2.33)	0.87 (0.53, 1.43)	0.91 (0.58, 1.43)	Reference	
$\ge$ 30.0 kg/m$^{2}$	1.66 (1.15, 2.40)	1.51 (1.09, 2.11)	1.25 (0.86, 1.83)	Reference	
Diabetes$^{d}$					0.005
no	1.25 (0.97, 1.60)	1.11 (0.82, 1.51)	0.93 (0.71, 1.22)	Reference	
yes	2.70 (1.80, 4.05)	2.06 (1.32, 3.21)	1.42 (0.91, 2.20)	Reference	
Chronic kidney disease$^{e}$					0.186
no	1.40 (1.05, 1.87)	1.37 (1.05, 1.79)	1.00 (0.73, 1.36)	Reference	
yes	1.90 (1.26, 2.89)	1.11 (0.72, 1.72)	1.14 (0.75, 1.75)	Reference	

a: Modified for gender, race, marital status (having a partner, others), poverty-to-income ratio (<1.3,1.3-2.9, $>=$3.0), education level (< high school, high school, > high school), alcohol intake (no, yes), smoke (never, former, now), moderate-vigorous physical activity, body mass index (<25.0, 25.0-29.9, $\ge$30.0), high waist circumference, phosphorus, calcium, serum iron, albumin, uric acid, urinary albumin/creatinine ratio, estimated glomerular filtration rate, serum 25-hydroxyvitamin D, DFE deficiency, Vitamin B12 deficiency, Iron intake deficiency, angina, heart attack, coronary heart disease, congestive heart failure, stroke, hypertension, hyperlipidemia, diabetes, chronic kidney diseases, cancer, and rheumatoid arthritis.

b: Modified for age, race, marital status (having a partner, others), poverty-to-income ratio(<1.3,1.3-2.9, $>=$3.0), education level (< high school, high school, > high school), alcohol intake (no, yes), smoke (never, former, now), moderate-vigorous physical activity, body mass index (<25.0, 25.0-29.9, $\ge$30.0), high waist circumference, phosphorus, calcium, serum iron, albumin, uric acid, urinary albumin/creatinine ratio, estimated glomerular filtration rate, serum 25-hydroxyvitamin D, DFE deficiency, Vitamin B12 deficiency, Iron intake deficiency, angina, heart attack, coronary heart disease, congestive heart failure, stroke, hypertension, hyperlipidemia, diabetes, chronic kidney diseases, cancer, and rheumatoid arthritis.

c: Modified for age, gender, race, marital status (having a partner, others), poverty-to-income ratio(<1.3,1.3-2.9, $>=$3.0), education level (< high school, high school, > high school), alcohol intake (no, yes), smoke (never, former, now), moderate-vigorous physical activity, high waist circumference, phosphorus, calcium, serum iron, albumin, uric acid, urinary albumin/creatinine ratio, estimated glomerular filtration rate, serum 25-hydroxyvitamin D, DFE deficiency, Vitamin B12 deficiency, Iron intake deficiency, angina, heart attack, coronary heart disease, congestive heart failure, stroke, hypertension, hyperlipidemia, diabetes, chronic kidney diseases, cancer, and rheumatoid arthritis.

d: Modified for age, gender, race, marital status (having a partner, others), poverty-to-income ratio(<1.3,1.3-2.9, $>=$3.0), education level (< high school, high school, > high school), alcohol intake (no, yes), smoke (never, former, now), moderate-vigorous physical activity, body mass index (<25.0, 25.0-29.9, $\ge$30.0), high waist circumference, phosphorus, calcium, serum iron, albumin, uric acid, urinary albumin/creatinine ratio, estimated glomerular filtration rate, serum 25-hydroxyvitamin D, DFE deficiency, Vitamin B12 deficiency, Iron intake deficiency, angina, heart attack, coronary heart disease, congestive heart failure, stroke, hypertension, hyperlipidemia, chronic kidney diseases, cancer, and rheumatoid arthritis.

e: Modified for age, gender, race, marital status (having a partner, others), poverty-to-income ratio(<1.3,1.3-2.9, $>=$3.0), education level (< high school, high school, > high school), alcohol intake (no, yes), smoke (never, former, now), moderate-vigorous physical activity, body mass index (<25.0, 25.0-29.9, $\ge$30.0), high waist circumference, phosphorus, calcium, serum iron, albumin, uric acid, urinary albumin/creatinine ratio, estimated glomerular filtration rate, serum 25-hydroxyvitamin D, DFE deficiency, Vitamin B12 deficiency, Iron intake deficiency, angina, heart attack, coronary heart disease, congestive heart failure, stroke, hypertension, hyperlipidemia, diabetes, cancer, and rheumatoid arthritis.

Abbreviations: *OR* odd ratio, *CI* confidence interval

## Discussion

This study is the first to examine the association between anemia and serum Klotho levels using a population-based, nationally representative sample of middle-aged and elderly people. Our results show that low levels of serum Klotho are associated with an increased incidence of anemia, a reduction in hemoglobin levels and red blood cell counts, and an increase in the width of the red blood cell distribution.

The prevalence of anemia increases rapidly after age 50, with over 20% of people developing anemia at $>=$85 years of age [12]. The NHANES III study categorized anemia studies as deficiencies in hematopoietic raw materials, renal insufficiency, chronic inflammation, unexplained anemia. One-third of anemia in the elderly is classified as unexplained and studies have found that such anemia is characterized by low EPO levels, low inflammatory factors, and low lymphocyte counts [13].

Klotho was first identified as an anti-aging protein. Later studies gradually revealed that CKD is characterised by the presence of Klotho deficiency. In mice and humans with KLOTHO missense mutations, elevated serum 1,25(OH)2D3 levels and impaired FGF23 signalling were seen, manifested by altered phosphate homeostasis and subsequent marked translocation calcification [14, 15]. In 2014, Lindsay M. Coe et al. found that Klotho mutant mice with elevated vitamin D activity had a severely disturbed hematopoietic environment, suppressing HIF and EPO in bone, with the same prenatal and postnatal hematopoietic disturbances [8]. Inflammatory stimulation of mice in early to middle age results in eventual peripheral haemocytopenia, bone marrow cytopenia, and build-up of bone marrow adipocytes, which together constitute typical features of hematopoiesis in elderly humans [16]. Aging and obesity lead to an increase in bone marrow adiposity, which activates inflammatory vesicles and thus negatively regulate B and T lymphocyte production. Previous studies have shown that KLOTHO inactivation is manifested by a reduction in the bone marrow and peripheral blood B lymphocytes [17].

Besides, in an observational case-control study that included 40 thalassaemia patients and 41 healthy controls, there was no correlation between iFGF23 and Klotho concentrations and serum ferritin levels after controlling for age or BMI [18]. Interestingly, a current analysis of data obtained from the NHANES study found that increased serum Klotho concentrations were significantly associated with higher vitamin B12 concentrations [19]. Klotho may also increase the risk of anemia by affecting the reduction of anemia-related factors.

Furthermore, Our stratified analysis found that low levels of serum Klotho were associated with a higher risk of developing anemia more significantly in men and diabetic patients. A study using a sample of 3750 men aged 40-79 years in the USA found a positive correlation between total testosterone and S-Klotho levels [20]. The role of androgens in stimulating erythropoiesis has been well established since the 20th century, and the testosterone produced by the testes is the main circulating androgen in men. Androgens were the only way to treat men with chronic kidney disease anemia before the advent of EPO [21]. Although the exact mechanism by which testosterone affects blood cells remains unclear, it is now believed that testosterone can act directly on the bone marrow to stimulate erythropoiesis by increasing EPO, inhibiting iron-regulating hormones and increasing the expression of iron transport proteins [22]. Anemia is also common in diabetics and the cause is multifactorial and not well understood, often attributed to systemic inflammation, autoimmunity, renal insufficiency, and lack of hematopoietic material. In addition, Mathis Grossmann, et al. found that men with type 2 diabetes and insulin resistance were more likely to have reduced testosterone (either total or free testosterone) [23]. Low levels of soluble Klotho have been shown in mouse studies to induce insulin resistance and to increase hepatic gluconeogenesis and hepatocyte lipid accumulation in T2D. In 2011 Ruifeng Teng et al. were the first to demonstrate that mice lacking erythropoietin receptors in non-hematopoietic tissues were more prone to increased white adiposity, increased inflammation, reduced energy expenditure, and insulin resistance compared to wild-type mice [24]. This may be highly suggestive of an effect of Klotho on the hematopoietic system not only through EPO but also possibly through effects on energy metabolism and sex hormones.

Stratified analysis showed that the relationship between anemia and serum Klotho was not altered by the presence or absence of chronic kidney disease. Previous studies in small samples have shown that low expression of $\alpha$-Klotho is associated with the development of anemia in patients with chronic kidney disease and is also involved in activating the HIF signaling pathway, increasing serum EPO and iron levels [25]. It has also been concluded that alpha-Klotho administration has no effect on EPO levels or hemoglobin in mice with CKD anemia [26]. This may be related to the dose and duration of Klotho maintenance treatment. As our research has shown an association with anemia, hemoglobin levels, red blood cell levels and the red cell distribution width at Klotho Q1 (496.7 - 613.9 pg/mL), Q2 (690.9 - 763.4 pg/mL). Again in the segmented regression it was indicated that the risk of anemia was reduced when Klotho<9.746/100 pg/mL. When Klotho is greater than 9.746/100 pg/mL, there may not be a relationship with the risk of anemia development. A recent study by Changhong Du et al. found that renal soluble Klotho can act directly on hematopoietic stem cells and that Klotho is involved in the regulation of bone marrow hematopoiesis by inhibiting SLC20A1-mediated inorganic phosphate uptake by hematopoietic stem cells [27]. It is evident that the interaction between the kidney and bone marrow hematopoietic stem cells goes beyond the role that can be explained by erythropoietin.

Our results should be considered in the context of several limitations. First, due to the retrospective, observational study design, the causal relationship between anemia and serum Klotho could not be fully assessed. Second, no data are currently available concerning the value of serum Klotho in young subjects. In addition to this, Klotho may increase the risk of anemia by reducing anemia-related factors, so more clinical trials are needed to explore the relationship between anemia factors and Klotho. Finally, it is beyond the scope of this study to explore the mechanisms between anemia and serum Klotho, and we lack data on FGF23 and EPO to further explore the mechanisms involved.

In conclusion, in our study, it is suggested that high levels of Klotho have a protective effect against anemia in middle age and that this effect is more pronounced in men and in diabetes. The interaction between kidney-secreted Klotho and bone marrow hematopoietic stem cells is complex and more research is needed to verify the relationship.

## Supplementary Information

**Additional file 1.** Baseline characteristics of the study participants from NHANES 2007-2016, weighted.

**Additional file 2.** Relationship between anemia and S-Klotho (pg/mL) in 3 models, weighted.

**Additional file 3.** Threshold effects of S-Klotho (per 100 pg/mL) for anemia were analyzed in NHANES2007-2016 using a two-piece regression model.

**Additional file 4.** Subgroup analyses for the association between anemia and quartiles of S-Klotho (pg/mL)among the study participants, weighted.

## Data Availability

All data analysed in this study are publicly available at the National Centre for Health Statistics: https://www.cdc.gov/nchs/nhanes/.

## Abbreviations

Klotho: *α*-Klotho
NHANES: National Health and Nutrition Examination Survey
FGF23: Fibroblast growth factor 23
HSC: Hematopoietic stem cells
HIF: Hypoxia-inducible factor
NCHS: National Center for Health Statistics
PIR: Poverty income ratio
METs: Metabolic Equivalents
eGFR: estimated glomerular filtration rate
BMI: Body mass index
hb: Hemoglobin
EPO: Erythropoietin
DFE: Folate as dietary folate equivalents
IQR: Interquartile range
