# Supplementary material for: Association between anemia and serum Klotho in middle-aged and older adults
Source: BMC Nephrol. 2023 Feb 16;24:38. doi: 10.1186/s12882-023-03081-w (PMC9933285; doi:10.1186/s12882-023-03081-w)
Supplement: Supplementary file 1 — Additional file 1. Baseline characteristics of the study participants from NHANES 2007-2016, weighted. [file 12882_2023_3081_MOESM1_ESM.docx]

**Supplementary Table 1. Relationship between hemoglobin(g/dL) and S-Klotho (pg/mL) in 3 models, weighted.**

| Exposure | Model 1 | | Model 2 | | Model 3 | |
| --- | --- | --- | --- | --- | --- | --- |
|  | Beta(95% CI) | *P*-value | Beta(95%CI) | *P*-value | Beta(95% CI) | *P*-value |
| **S-Klotho categories** | | |  | |  | |
| Quartile 1  (496.7 ,613.9) | -0.2 (-0.3, -0.2) | <0.001 | -0.3 (-0.3,-0.2) | <0.001 | -0.3 (-0.4,-0.2) | <0.001 |
| Quartile 2  (690.9 ,763.4) | -0.1 (-0.2, -0.0) | 0.004 | -0.1 (-0.2,-0.1) | <0.001 | -0.2 (-0.2,-0.1) | <0.001 |
| Quartile 3  (840.8 ,935.5) | -0.0 (-0.1, 0.1) | 0.945 | -0.0 (-0.1,0.0) | 0.455 | -0.0 (-0.1, 0.0) | 0.118 |
| Quartile 4  (1060.8 ,1330.9) | Reference | | Reference | | Reference | |

Model 1: Modified for age, gender, and race.

Model 2: Modified for age, gender, race, marital status(having a partner,others), poverty-to-income ratio(<1.3,1.3-2.9,>=3.0), education level (< high school, high school,> high school), alcohol intake (no, yes), smoke (never, former,now), moderate-vigorous physical activity, body mass index (<25.0 ,25.0-29.9,≥30.0), and high waist circumference.

Model 3: Modified for age, gender, race, marital status(having a partner,others), poverty-to-income ratio(<1.3,1.3-2.9,>=3.0), education level (< high school, high school,> high school), alcohol intake (no, yes), smoke (never, former,now), moderate-vigorous physical activity, body mass index (<25.0 ,25.0-29.9,≥30.0), high waist circumference, phosphorus, calcium, serum iron, albumin, uric acid, urinary albumin/creatinine ratio, estimated glomerular filtration rate, serum 25-hydroxyvitamin D, DFE deficiency, Vitamin B12 deficiency, Iron intake deficiency, angina, heart attack, coronary heart disease, congestive heart failure, stroke, hypertension, hyperlipidemia, diabetes, chronic kidney diseases, cancer, and rheumatoid arthritis

Abbreviations: CI, confidence interval.

.
