# Supplementary material for: Association between anemia and serum Klotho in middle-aged and older adults
Source: BMC Nephrol. 2023 Feb 16;24:38. doi: 10.1186/s12882-023-03081-w (PMC9933285; doi:10.1186/s12882-023-03081-w)
Supplement: Supplementary file 3 — Additional file 3. Threshold effects of S-Klotho (per 100 pg/mL) for anemia were analyzed in NHANES2007-2016 using a two-piece regression model. [file 12882_2023_3081_MOESM3_ESM.docx]

**Supplementary Table 3. Relationship between Red cell distribution width and S-Klotho (pg/mL) in 3 models, weighted.**

| Exposure | Model 1 | | Model 2 | | Model 3 | |
| --- | --- | --- | --- | --- | --- | --- |
|  | Beta(95% CI) | *P*-value | Beta(95% CI) | *P*-value | Beta(95% CI) | *P*-value |
| **S-Klotho categories** | | |  | |  | |
| Quartile 1  (496.7 ,613.9) | 0.1 (0.1, 0.2) | <0.001 | 0.1 (0.1, 0.2) | <0.001 | 0.1 (0.1, 0.2) | <0.001 |
| Quartile 2  (690.9 ,763.4) | 0.1 (0.0, 0.1) | 0.003 | 0.1 (0.0, 0.1) | 0.013 | 0.1 (0.0, 0.2) | 0.005 |
| Quartile 3  (840.8 ,935.5) | 0.0 (-0.0, 0.1) | 0.255 | 0.0 (-0.0, 0.1) | 0.305 | 0.1 (-0.0, 0.1) | 0.122 |
| Quartile 4  (1060.8 ,1330.9) | Reference | | Reference | | Reference | |

Model 1: Modified for age, gender, and race.

Model 2: Modified for age, gender, race, marital status(having a partner,others), poverty-to-income ratio(<1.3,1.3-2.9,>=3.0), education level (< high school, high school,> high school), alcohol intake (no, yes), smoke (never, former,now), moderate-vigorous physical activity, body mass index (<25.0 ,25.0-29.9,≥30.0), and high waist circumference.

Model 3: Modified for age, gender, race, marital status(having a partner,others), poverty-to-income ratio(<1.3,1.3-2.9,>=3.0), education level (< high school, high school,> high school), alcohol intake (no, yes), smoke (never, former,now), moderate-vigorous physical activity, body mass index (<25.0 ,25.0-29.9,≥30.0), high waist circumference, phosphorus, calcium, serum iron, albumin, uric acid, urinary albumin/creatinine ratio, estimated glomerular filtration rate, serum 25-hydroxyvitamin D, DFE deficiency, Vitamin B12 deficiency, Iron intake deficiency, angina, heart attack, coronary heart disease, congestive heart failure, stroke, hypertension, hyperlipidemia, diabetes, chronic kidney diseases, cancer, and rheumatoid arthritis.

Abbreviations: CI, confidence interval.
