# Supplementary material for: Association between anemia and serum Klotho in middle-aged and older adults
Source: BMC Nephrol. 2023 Feb 16;24:38. doi: 10.1186/s12882-023-03081-w (PMC9933285; doi:10.1186/s12882-023-03081-w)
Supplement: Supplementary file 4 — Additional file 4. Subgroup analyses for the association between anemia and quartiles of S-Klotho (pg/mL)among the study participants, weighted. [file 12882_2023_3081_MOESM4_ESM.pdf]

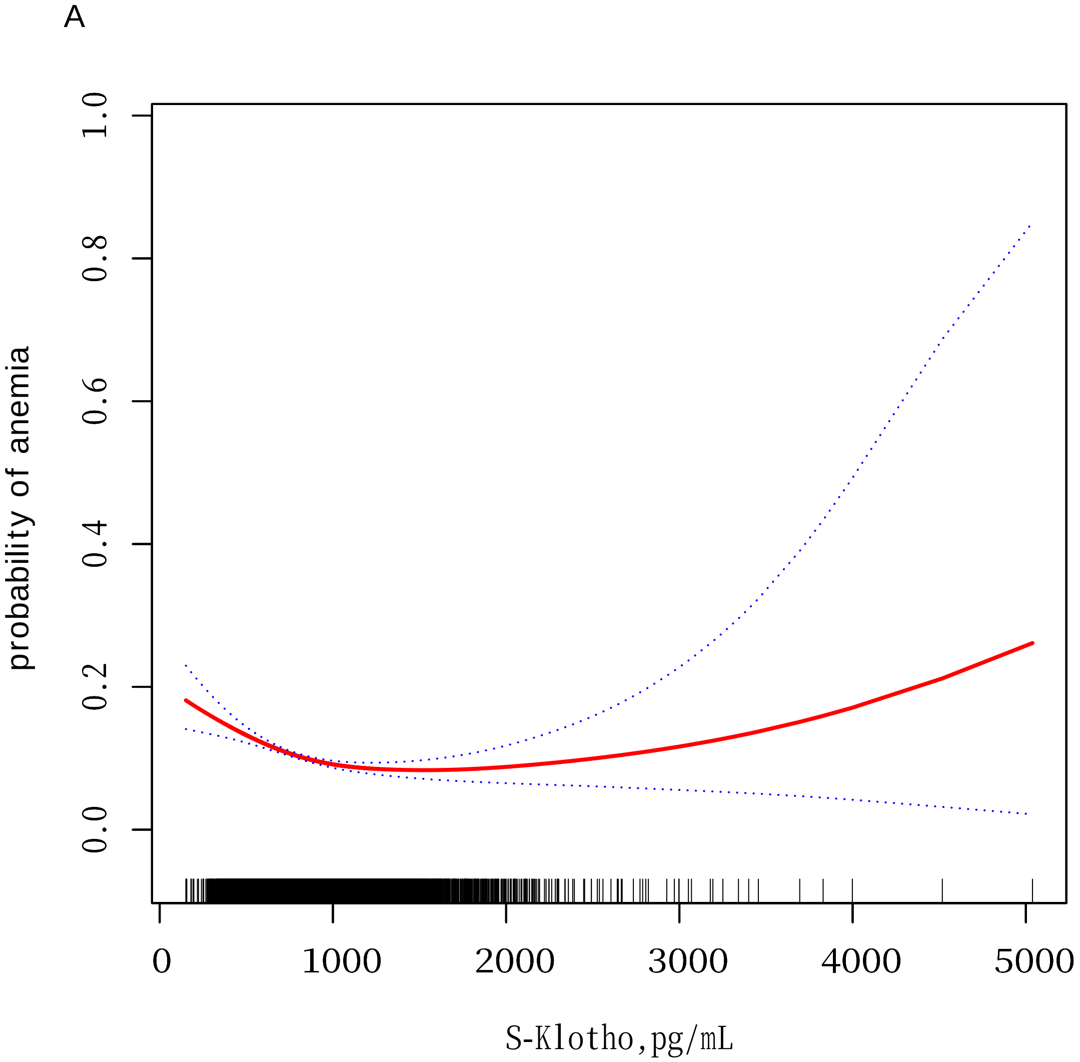
A
probability of anemia
0.0
0.2
0.4
0.6
0.8
1.0
0
1000
2000
3000
4000
5000
S-Klotho,pg/mL

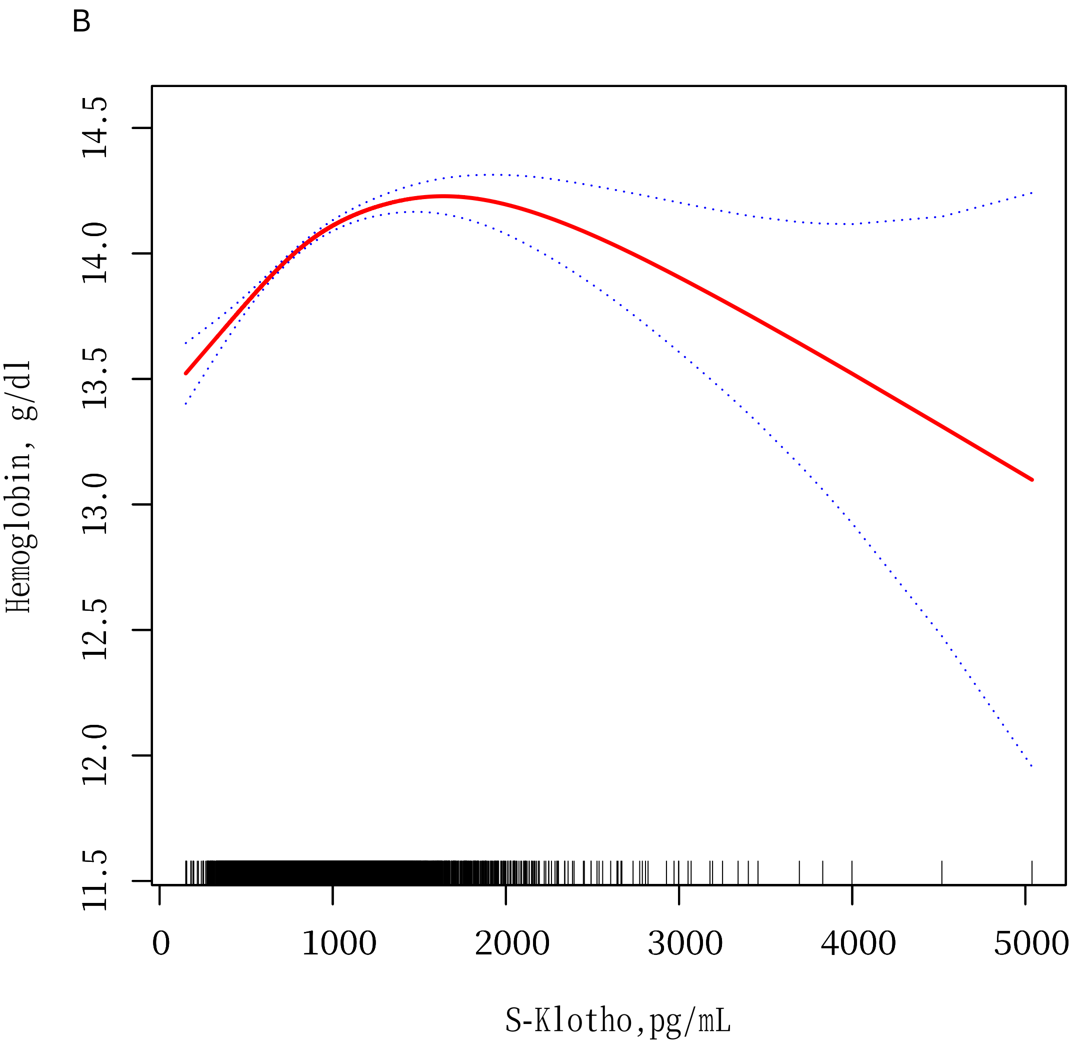
B
14.5
14.0
13.5
13.0
12.5
12.0
11.5
Hemoglobin, g/dl
0
1000
2000
3000
4000
5000
S-Klotho,pg/mL

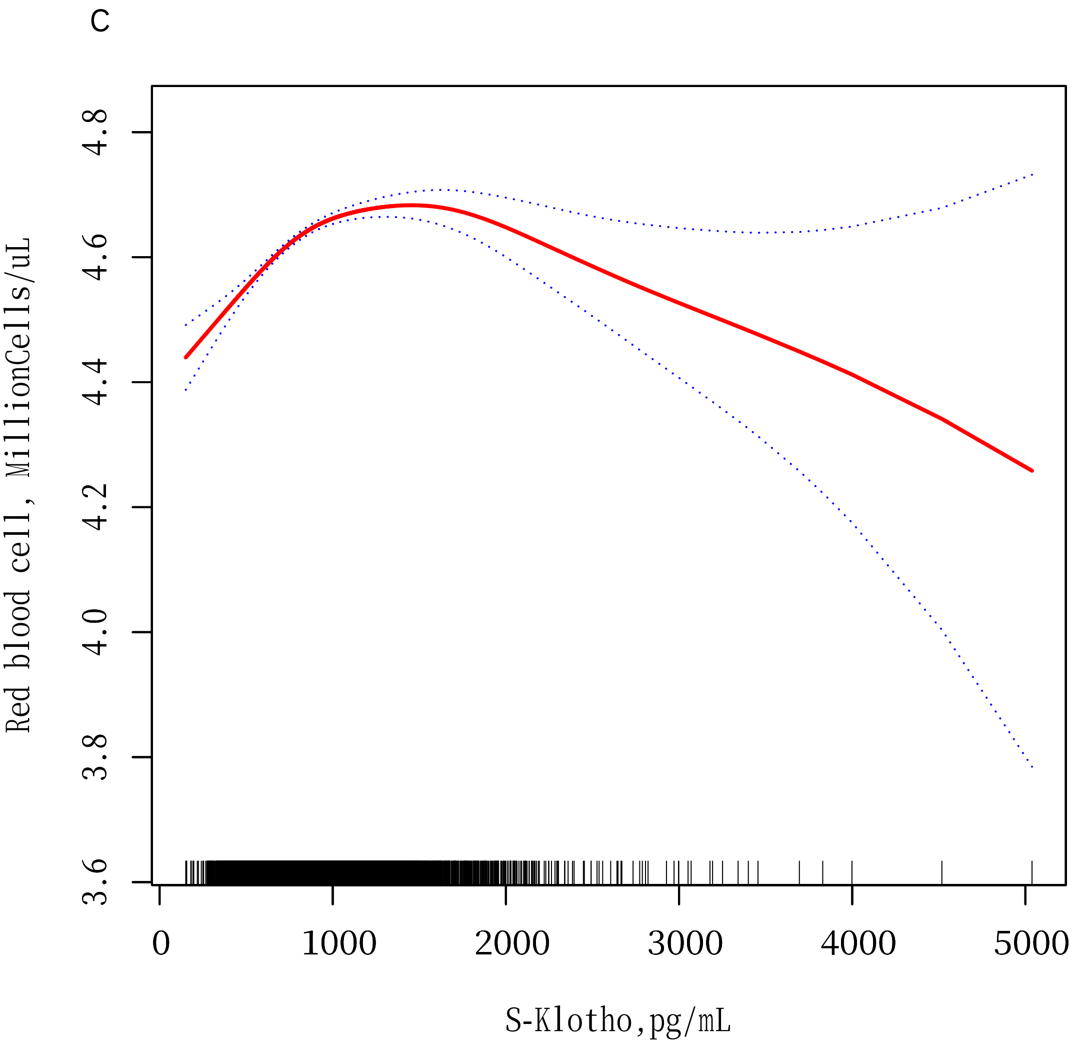
C
4.8
4.6
4.4
4.2
4.0
3.8
3.6
Red blood cell, MillionCells/uL
0
1000
2000
3000
4000
5000
S-Klotho,pg/mL

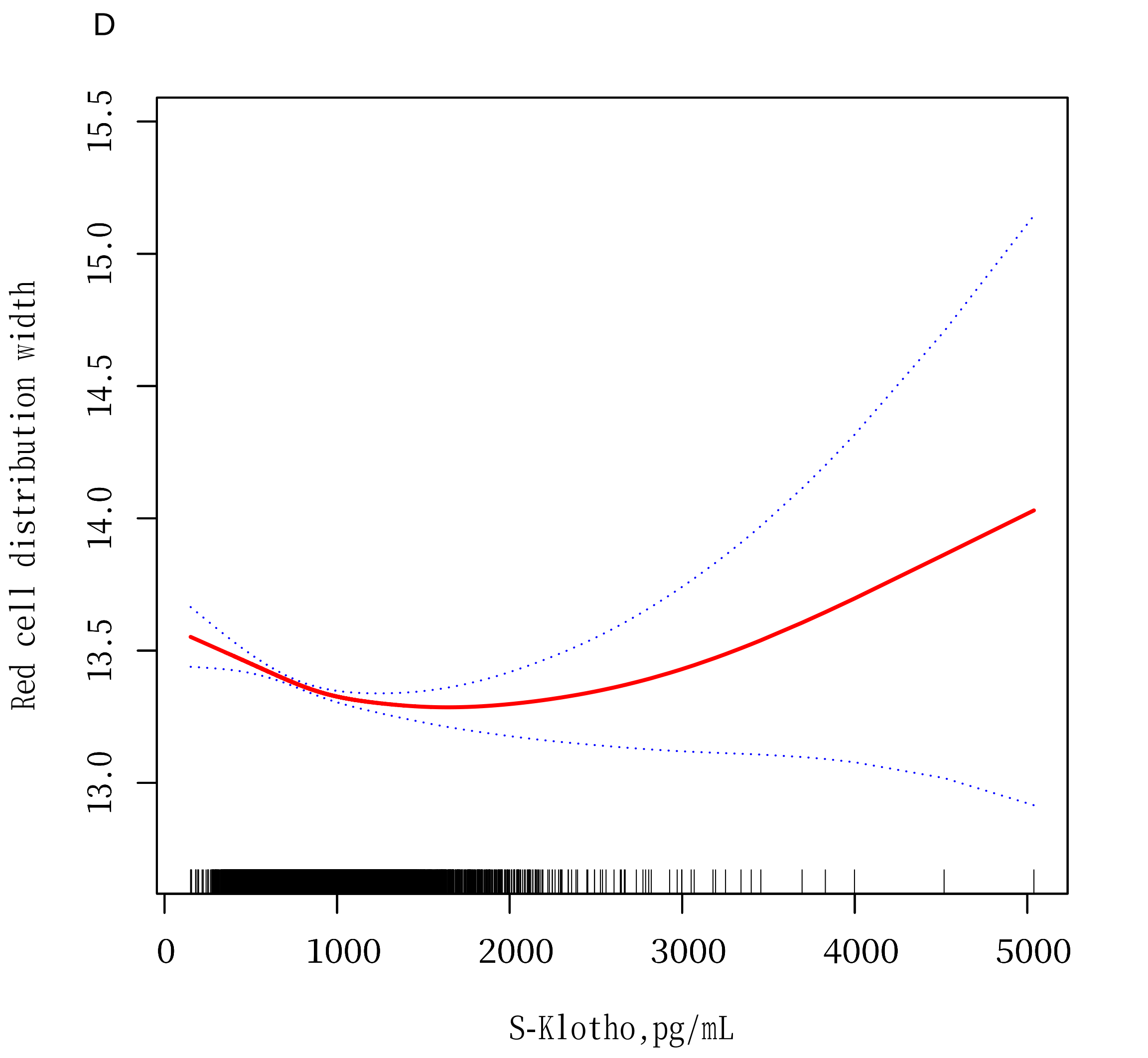
D
Red cell distribution width
13.0
13.5
14.0
14.5
15.0
15.5
0
1000
2000
3000
4000
5000
S-Klotho,pg/mL

Supplementary figure 1: The fitted smooth curve showed the association between anemia-related indicators and S-Klotho after adjusting the relative confounding factors (age, gender, race, marital status(having a partner, others), poverty-to-income ratio(<1.3,1.3-2.9,>=3.0), education level (< high school, high school,> high school), alcohol intake (no, yes), smoke (never, former,now), moderate-vigorous physical activity, body mass index (<25.0 ,25.0-29.9,≥30.0), high waist circumference, phosphorus, calcium, serum iron, albumin, uric acid, urinary albumin/creatinine ratio, estimated glomerular filtration rate, serum 25-hydroxyvitamin D, DFE deficiency, Vitamin B12 deficiency, Iron intake deficiency, angina, heart attack, coronary heart disease, congestive heart failure, stroke, hypertension, hyperlipidemia, diabetes, chronic kidney diseases, cancer, and rheumatoid arthritis.) between anemia-related indicators and S-Klotho concentrations among adults in NHANES 2007-2016. The area between the dotted lines represents the 95% confidence interval.

A. Non-linear relationships between provability of anemia and S-Klotho.

B. Non-linear relationships between hemoglobin(g/dL) and S-Klotho.

C. Non-linear relationships between red blood cells (MillionCells/uL) and S-Klotho.

D. Non-linear relationships between red cell distribution width and S-Klotho.
